# Blood urea nitrogen to albumin ratio is associated with cerebral small vessel diseases

**DOI:** 10.1038/s41598-024-54919-8

**Published:** 2024-02-23

**Authors:** Ki-Woong Nam, Hyung-Min Kwon, Han-Yeong Jeong, Jin-Ho Park, Kyungha Min

**Affiliations:** 1 Department of Neurology, Seoul National University College of Medicine, Seoul, Korea; 2 Department of Neurology, Seoul Metropolitan Government-Seoul National University Boramae Medical Center, 20 Boramae-ro 5-gil, Dongjak-gu, Seoul, 07061 South Korea; 3 Department of Family Medicine, Seoul National University College of Medicine and Seoul National University Hospital, 101 Daehak-ro, Jongno-gu, Seoul, 03080 South Korea

**Keywords:** Neurology, Neurological disorders

## Abstract

Blood urea nitrogen (BUN) to albumin ratio (BAR) is a comprehensive parameter that reflects renal, inflammatory, nutritional, and endothelial functions. BAR has been shown to be associated with various cancers, pneumonia, sepsis, and pulmonary and cardiovascular diseases; however, few studies have been conducted on its association with cerebrovascular diseases. In this study, we evaluated the association between BAR and cerebral small vessel disease (cSVD) in health check-up participants. We assessed consecutive health check-up participants between January 2006 and December 2013. For the cSVD subtype, we quantitatively measured the volume of white matter hyperintensity (WMH) and qualitatively measured the presence of lacune and cerebral microbleeds (CMBs). The BAR was calculated by dividing BUN by albumin as follows: BAR = BUN (mg/dl)/albumin (g/dl). A total of 3012 participants were evaluated. In multivariable linear regression analysis, BAR showed a statistically significant association with WMH volume after adjusting for confounders [*β* = 0.076, 95% confidence interval (CI): 0.027–0.125]. In multivariable logistic regression analyses, BAR was significantly associated with lacunes [adjusted odds ratio (aOR) = 1.20, 95% CI: 1.00–1.44] and CMBs (aOR = 1.28, 95% CI: 1.06–1.55). BAR was associated with all types of cSVD in the health check-up participants.

## Introduction

Cerebral small vessel diseases (cSVD)s represent chronic subclinical pathologies including various subtypes such as white matter hyperintensity (WMH), lacunes of presumed vascular origin, and cerebral microbleeds (CMBs)^1,2^. The increase in elderly population and development of brain imaging technology worldwide has increased the prevalence of cSVD, which is now commonly encountered in health check-ups^3^. Although the subtypes of cSVD are very different in appearance, they are usually found coexisting on brain magnetic resonance imaging (MRI)^4^. Therefore, the different subtypes of cSVDs are thought to occur via a common pathological mechanism^2,5^.

As a subclinical pathology, it has been known that cSVD does not cause symptoms independently. However, cSVD lesions can increase the risk of stroke and dementia, resulting in a more severe or poor prognosis^1,6,7^. To be precise, cSVD cannot be considered an intermediate stage of stroke. However, it would be clinically desirable if it could be prevented at the level of asymptomatic cSVD before subsequent severe cerebrovascular events occur. Therefore, many studies have been conducted in various areas to identify the risk factors and pathological mechanisms of cSVD (e.g., demographic, metabolic, inflammatory, and anthropometric areas)^8–11^. Moreover, recent studies have shown that the large burden of cSVD lesions themselves can cause symptoms such as dysphagia, cognitive impairment, and gait disturbance, thus, suggesting the importance of finding out the pathogenesis of cSVD^12^.

Blood urea nitrogen (BUN) to albumin ratio (BAR) is a comprehensive parameter that reflects renal, inflammatory, nutritional, and endothelial functions^13,14^. Since its first appearance, BAR has been shown to be significantly associated with various diseases, such as pneumonia, sepsis, chronic obstructive pulmonary disease, coronavirus disease-19, cancer, gastrointestinal bleeding, and cardiovascular disease^14–19^. However, the association of BAR with cerebrovascular disease has not been studied enough. In addition, studies related to BAR have mainly been conducted in emergency or acute-stage diseases (e.g., dehydration, bleeding, and low cardiac output) in which BUN elevation through renal reabsorption is likely to occur due to a decrease in circulating volume^16,18,20,21^. Nevertheless, considering the various characteristics of the conditions with high BUN and low albumin levels, BAR is thought to be involved in the development of chronic diseases.

In this study, we evaluated the association between BAR and cSVD in health check-up participants. In addition, analysis according to cSVD subtype and additional analysis using other biomarkers were conducted to determine the pathological mechanism between BAR and cSVD.

## Results

### General information of study population

A total of 3012 health check-up participants were evaluated (median age: 56 ± 9 years, male sex: 55.7%). The mean WMH volume was 2.60 ± 6.23 mL, and the prevalence of lacunes and CMBs was 220 (7.3%) and 127 (4.2%), respectively. Detailed baseline characteristics of the study population are presented in Table 1.

**Table 1:** Baseline characteristics of the cohort (n = 3012).

	Total
Age, y [IQR]	56 [50–63]
Sex, male, n (%)	1678 (55.7)
Body mass index, kg/m^2^ [IQR]	24.04 [22.13–25.93]
Hypertension, n (%)	760 (25.2)
Diabetes, n (%)	452 (15.0)
Hyperlipidemia, n (%)	456 (15.1)
Ischemic heart disease, n (%)	102 (3.4)
Current smoking, n (%)	555 (18.4)
Systolic blood pressure, mmHg [IQR]	126 [115–136]
Diastolic blood pressure, mmHg [IQR]	75 [69–83]
Fasting glucose, mg/dl [IQR]	92 [85–101]
Total cholesterol, mg/dl [IQR]	198 [174–223]
Low-density lipoprotein cholesterol, mg/dl [IQR]	125 [101–148]
High-density lipoprotein cholesterol, mg/dl [IQR]	53 [44–63]
Triglyceride, mg/dl [IQR]	101 [74–146]
White blood cell counts, × 10^3^/μl [IQR]	5.33 [4.43–6.40]
High-sensitivity C-reactive protein, mg/dl [IQR]	0.04 [0.01–0.15]
Blood urea nitrogen, mg/dl [IQR]	14 [12–16]
Creatinine, mg/dl [IQR]	0.90 [0.76–1.02]
GFR (MDRD equation), ml/min/1.73 m^2^ [IQR]	78.20 [69.50–88.67]
Albumin, g/dl [IQR]	4.4 [4.3–4.6]
BUN/albumin ratio [IQR]	3.04 [2.56–3.62]
WMH volume, mL [IQR]	1.05 [0.20–2.60]
Lacunes of presumed vascular origin, n (%)	220 (7.3)
Single	171 (5.7)
Multiple	32 (1.1)
Cerebral microbleeds, n (%)	127 (4.2)
Single	88 (2.9)
Multiple	39 (1.3)

*IQR* interquartile range, *GFR* glomerular filtration rate, *MDRD* Modification of Diet in Renal Disease study, *BUN* blood urea nitrogen, *WMH* white matter hyperintensity.

### Characteristics according to BAR tertiles

When comparing the baseline characteristics of participants according to BAR tertiles, subjects with high BAR were positively associated with age, male sex, body mass index (BMI), hypertension, diabetes, hyperlipidemia, fasting glucose, WBC counts, BUN, creatinine, high-sensitivity C-reactive protein (hs-CRP), WMH volume, lacunes, and CMBs. In contrast, current smoking, diastolic blood pressure (BP), and total cholesterol, triglyceride, glomerular filtration rate (GFR), and albumin levels were negatively associated with BAR (Table 2).

**Table 2:** Comparisons of baseline characteristics according to the BAR tertiles.

	BAR Tertile 1 < 2.73	BAR Tertile 2 2.73–3.40	BAR Tertile 3 > 3.40	*P*-value	*P*-trend
Number	1010	994	1008		
Blood urea nitrogen, mg/dl [IQR]	11 [10–12]	14 [13–14]	17 [16–19]	< 0.001	< 0.001
Albumin, g/dl [IQR]	4.5 [4.4–4.6]	4.5 [4.3–4.6]	4.4 [4.2–4.5]	< 0.001	< 0.001
Age, y [IQR]	53 [47–60]	56 [50–62]	59 [54–66]	< 0.001	< 0.001
Sex, male, n (%)	495 (49.0)	581 (58.5)	602 (59.7)	< 0.001	< 0.001
Body mass index, kg/m^2^ [IQR]	23.72 [21.84–25.60]	24.10 [22.24–26.12]	24.26 [22.38–26.27]	< 0.001	< 0.001
Hypertension, n (%)	224 (22.0)	244 (24.5)	292 (29.0)	0.002	< 0.001
Diabetes, n (%)	122 (12.1)	142 (14.3)	188 (18.7)	< 0.001	< 0.001
Hyperlipidemia, n (%)	128 (12.7)	159 (16.0)	169 (16.8)	0.024	0.010
Ischemic heart disease, n (%)	26 (2.6)	32 (3.2)	44 (4.4)	0.079	0.026
Current smoking, n (%)	206 (20.4)	198 (19.9)	151 (15.0)	0.002	0.002
Systolic blood pressure, mmHg [IQR]	126 [115–138]	126 [115–135]	126 [116–136]	0.523	0.709
Diastolic blood pressure, mmHg [IQR]	76 [70–85]	75 [69–82]	75 [69–83]	0.012	0.006
Fasting glucose, mg/dl [IQR]	91 [85–99]	92 [85–102]	92 [85–104]	0.040	0.020
Total cholesterol, mg/dl [IQR]	199 [175–224]	199 [176–224]	196 [172–220]	0.049	0.097
LDL cholesterol, mg/dl [IQR]	125 [101–148]	127 [105–149]	123 [99–147]	0.119	0.576
HDL cholesterol, mg/dl [IQR]	53 [44–63]	53 [44–63]	53 [44–63]	0.948	0.747
Triglyceride, mg/dl [IQR]	108 [78–152]	100 [73–148]	96 [72–136]	< 0.001	< 0.001
White blood cell counts, × 10^3^/μl [IQR]	5.25 [4.33–6.35]	5.28 [4.42–6.31]	5.47 [4.60–6.55]	0.001	0.001
Creatinine, mg/dl [IQR]	0.87 [0.74–1.00]	0.90 [0.77–1.03]	0.91 [0.78–1.07]	< 0.001	< 0.001
GFR (MDRD equation), ml/min/1.73 m^2^ [IQR]	79.95 [71.75–90.71]	78.24 [69.90–87.79]	76.75 [67.15–87.25]	< 0.001	< 0.001
High-sensitivity CRP, mg/dl [IQR]	0.04 [0.01–0.14]	0.04 [0.01–0.14]	0.06 [0.01–0.17]	0.001	0.003
WMH volume, mL [IQR]	0.80 [0.10–2.20]	1.00 [0.20–2.57]	1.41 [0.40–3.24]	< 0.001	< 0.001
Lacunes, n (%)	61 (6.0)	57 (5.7)	102 (10.1)	< 0.001	< 0.001
Cerebral microbleeds, n (%)	27 (2.7)	42 (4.2)	58 (5.8)	0.003	0.001

*BAR* blood urea nitrogen/albumin ratio, *IQR* interquartile range, *LDL* low-density lipoprotein, *HDL* high-density lipoprotein, *GFR* glomerular filtration rate, *MDRD* Modification of Diet in Renal Disease study, *CRP* C-reactive protein, *WMH* white matter hyperintensity.

### Association between BAR and each type of cSVD pathology

In our data, WMH volume was related to age, hypertension, diabetes, current smoking, systolic and diastolic BP, fasting glucose, low-density lipoprotein (LDL) cholesterol, white blood cell (WBC) counts, BUN, creatinine, GFR, albumin, and BAR. In the multivariable linear regression analysis, BAR was significantly associated with WMH volume after adjusting for confounders [*β* = 0.076, 95% confidence interval (CI): 0.027–0.125]. Age (*β* = 0.045, 95% CI: 0.041–0.050), BMI (*β* = − 0.020, 95% CI: − 0.034 to − 0.007), hypertension (*β* = 0.135, 95% CI: 0.041–0.230), systolic BP (*β* = 0.006, 95% CI: 0.004–0.009), and WBC counts (*β* = 0.045, 95% CI: 0.020–0.071) were also associated with WMH volume (Table 3).

**Table 3:** Univariate and multivariable linear regression analyses to evaluate the association between possible predictors and white matter hyperintensity volume.

	Univariable analysis		Multivariable analysis	
	B (95% CI)	*P* value	B (95% CI)	*P* value
Age	0.052 (0.049 to 0.056)	< 0.001	0.045 (0.041 to 0.050)	< 0.001
Sex, male	0.020 (− 0.059 to 0.099)	0.624	…	…
Body mass index	− 0.003 (− 0.016 to 0.010)	0.623	− 0.020 (− 0.034 to − 0.007)	0.003
Hypertension	0.422 (0.333 to 0.511)	< 0.001	0.135 (0.041 to 0.230)	0.005
Diabetes	0.353 (0.243 to 0.462)	< 0.001	0.024 (− 0.111 to 0.159)	0.724
Hyperlipidemia	0.037 (− 0.073 to 0.147)	0.507	…	…
Ischemic heart disease	0.104 (− 0.114 to 0.321)	0.351	…	…
Current smoking	− 0.197 (− 0.299 to − 0.096)	< 0.001	0.041 (− 0.065 to 0.148)	0.448
Systolic blood pressure	0.010 (0.008 to 0.013)	< 0.001	0.006 (0.004 to 0.009)	< 0.001
Diastolic blood pressure	0.007 (0.003 to 0.011)	< 0.001	…	…
Fasting glucose*	0.604 (0.403 to 0.805)	< 0.001	0.059 (− 0.194 to 0.312)	0.648
Total cholesterol	− 0.001 (− 0.002 to 0.000)	0.070	…	…
LDL cholesterol	− 0.001 (− 0.002 to 0.000)	0.046	0.000 (− 0.001 to 0.001)	0.520
HDL cholesterol	− 0.001 (− 0.004 to 0.002)	0.571	…	…
Triglyceride*	0.041 (− 0.038 to 0.119)	0.310	…	…
White blood cell counts	0.047 (0.024 to 0.071)	< 0.001	0.045 (0.020 to 0.071)	< 0.001
High-sensitivity CRP*	0.013 (− 0.013 to 0.039)	0.334	…	…
Blood urea nitrogen	0.047 (0.037 to 0.058)	< 0.001	…	…
Creatinine*	0.258 (0.076 to 0.440)	0.005	…	…
GFR (MDRD equation)	− 0.008 (− 0.010 to − 0.005)	< 0.001	0.001 (− 0.002 to 0.003)	0.661
Albumin	− 0.458 (− 0.617 to − 0.300)	< 0.001	…	…
BUN/albumin ratio	0.231 (0.185 to 0.276)	< 0.001	0.076 (0.027 to 0.125)	0.002

*LDL* low-density lipoprotein, *HDL* high-density lipoprotein, *CRP* C-reactive protein, *GFR* glomerular filtration rate, *MDRD* Modification of Diet in Renal Disease study, *BUN* blood nitrogen urea.

*These variables were transformed into a log scale.

In multivariable logistic regression analysis, BAR was significantly associated with lacunes [adjusted odds ratio (aOR) = 1.20, 95% CI: 1.00–1.44] and CMB (aOR = 1.28, 95% CI: 1.06–1.55) after adjusting for confounders. Additionally, lacunes showed a statistically significant association with age (aOR = 1.10, 95% CI: 1.07–1.12, Table 4), and CMBs were significantly associated with age (aOR = 1.05, 95% CI: 1.03–1.08), hypertension (aOR = 1.55, 95% CI: 1.06–2.28), and systolic BP (aOR = 1.02, 95% CI: 1.00–1.03, Table 5).

**Table 4:** Univariate and multivariable logistic regression analyses to evaluate the association between possible predictors and lacunes.

	Univariate		Multivariable	
	OR [95% CI]	*P* value	Adjusted OR [95% CI]	*P* value
Age	1.09 [1.08–1.11]	< 0.001	1.10 [1.07–1.12]	< 0.001
Sex, male	1.03 [0.78–1.36]	0.839	…	…
Body mass index	1.01 [0.97–1.05]	0.710	0.98 [0.93–1.04]	0.554
Hypertension	2.06 [1.55–2.74]	< 0.001	1.19 [0.83–1.71]	0.351
Diabetes	1.96 [1.41–2.71]	< 0.001	1.34 [0.82–2.20]	0.243
Hyperlipidemia	0.88 [0.59–1.31]	0.519	…	…
Ischemic heart disease	1.24 [0.62–2.49]	0.549	…	…
Current smoking	0.86 [0.59–1.24]	0.413	…	…
Systolic blood pressure	1.02 [1.01–1.03]	< 0.001	1.01 [1.00–1.02]	0.073
Diastolic blood pressure	1.02 [1.01–1.03]	0.003	…	…
Fasting glucose*	2.63 [1.42–4.86]	0.002	1.32 [0.50–3.48]	0.579
Total cholesterol	1.00 [0.99–1.00]	0.025	…	…
LDL cholesterol	1.00 [0.99–1.00]	0.026	1.00 [0.99–1.00]	0.651
HDL cholesterol	0.99 [0.98–1.00]	0.117	…	…
Triglyceride*	1.30 [0.99–1.69]	0.058	1.31 [0.91–1.89]	0.146
WBC counts	1.06 [0.98–1.14]	0.175	…	…
High-sensitivity CRP*	1.09 [1.00–1.19]	0.061	1.01 [0.90–1.13]	0.841
Blood urea nitrogen	1.08 [1.04–1.11]	< 0.001	…	…
Creatinine*	2.41 [1.28–4.53]	0.006	…	…
GFR (MDRD equation)	0.98 [0.97–0.99]	< 0.001	1.00 [0.99–1.01]	0.930
Albumin	0.31 [0.18–0.54]	< 0.001	…	…
BUN/albumin ratio	1.45 [1.26–1.67]	< 0.001	1.20 [1.00–1.44]	0.049

*OR* odds ratio, *LDL* low-density lipoprotein, *HDL* high-density lipoprotein, *WBC* white blood cell, *CRP* C-reactive protein, *GFR* glomerular filtration rate, *MDRD* Modification of Diet in Renal Disease study, *BUN* blood urea nitrogen.

*These variables were transformed into a log scale.

**Table 5:** Univariate and multivariable logistic regression analyses to evaluate the association between possible predictors and cerebral microbleeds.

	Univariate		Multivariable	
	OR [95% CI]	*P* value	Adjusted OR [95% CI]	*P* value
Age	1.07 [1.05–1.09]	< 0.001	1.05 [1.03–1.08]	< 0.001
Sex, male	1.24 [0.86–1.78]	0.255	…	…
Body mass index	1.00 [0.94–1.06]	0.939	0.96 [0.90–1.02]	0.159
Hypertension	2.13 [1.48–3.07]	< 0.001	1.55 [1.06–2.28]	0.027
Diabetes	1.49 [0.95–2.31]	0.080	0.98 [0.62–1.56]	0.929
Hyperlipidemia	0.92 [0.56–1.54]	0.756	…	…
Ischemic heart disease	1.18 [0.47–2.95]	0.726	…	…
Current smoking	0.58 [0.34–1.01]	0.052	0.77 [0.43–1.36]	0.360
Systolic blood pressure	1.02 [1.01–1.03]	0.001	1.02 [1.00–1.03]	0.013
Diastolic blood pressure	1.01 [1.00–1.03]	0.194	…	…
Fasting glucose*	1.69 [0.73–3.91]	0.222	…	…
Total cholesterol	1.00 [0.99–1.00]	0.780	…	…
LDL cholesterol	1.00 [1.00–1.01]	0.942	…	…
HDL cholesterol	0.99 [0.98–1.00]	0.093	0.99 [0.97–1.00]	0.055
Triglyceride*	0.93 [0.65–1.33]	0.704	…	…
WBC counts	1.08 [0.98–1.19]	0.133	…	…
High-sensitivity CRP*	1.06 [0.95–1.19]	0.297	…	…
Blood urea nitrogen	1.10 [1.05–1.14]	< 0.001	…	…
Creatinine*	1.99 [0.88–4.50]	0.099	…	…
GFR (MDRD equation)	0.99 [0.98–1.00]	0.159	1.00 [0.99–1.02]	0.594
Albumin	0.76 [0.37–1.55]	0.447	…	…
BUN/albumin ratio	1.50 [1.26–1.79]	< 0.001	1.28 [1.06–1.55]	0.012

*OR* odds ratio, *LDL* low-density lipoprotein, *HDL* high-density lipoprotein, *WBC* white blood cell, *CRP* C-reactive protein, *GFR* glomerular filtration rate, *MDRD* Modification of Diet in Renal Disease study, *BUN* blood urea nitrogen.

*These variables were transformed into a log scale.

**Figure 1:**
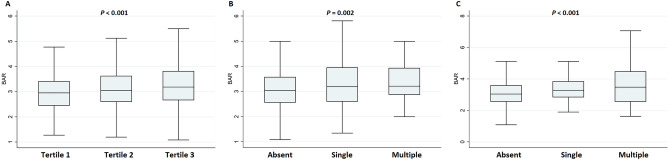
The relationship between blood urea nitrogen to albumin ratio and each subtype of cerebral small vessel disease. The blood urea nitrogen to albumin ratio showed a significant association with white matter hyperintensity volume tertile (*P* for trend < 0.001), lacunes (*P* for trend = 0.002), and cerebral microbleeds (*P* for trend < 0.001) in a positive quantitative manner.

When evaluating the quantitative relationship between BAR value and disease burden of each cSVD subtype, WMH tertile (*P* for trend < 0.001) and number of lacunes (*P* for trend = 0.002) and CMBs (*P* for trend < 0.001) all showed a positive quantitative relationship with BAR (Fig. 1).

### Comparison of strength of association of BAR and related variables with cSVD

Compared with BAR, each of BUN, albumin, creatinine, and creatinine-to-albumin ratio did not show a closer association with the cSVD subtype. BUN showed a statistically significant association only with WMH volume and CMB, while the creatinine-to-albumin ratio was significantly associated with WMH volume and lacunes. Although albumin and creatinine levels showed a statistical tendency, no significant level of association was observed with any cSVD subtype (Supplementary Table 1).

## Discussion

The present study showed that BAR was significantly associated with cSVD in health check-up participants. BAR was significantly associated with all types of cSVD and even showed a positive quantitative relationship. Therefore, BAR can be considered a common risk factor that influences different cSVD pathologies. In contrast, BUN showed significant association only with some of the cSVD subtypes, while albumin showed only statistical tendency without any significance. Consequently, the synergistic effect of high BUN and low albumin levels appears to be an indicator that can distinguish high-risk individuals with a high incidence of cSVD.

An exact pathological mechanism describing the close association between BAR and cSVD remains unknown. In particular, because BAR has been studied only in emergency or acute diseases, it is difficult to find a link between BAR and chronic diseases like cSVD, through previous studies^16,19,21^. The association between BAR and cSVD may be elucidated based on the characteristics of the two components that make up the BAR. First, we would like to look at it from the perspective of BUN. BUN is an indirect indicator of renal function and can indicate the likelihood of developing small vessel disease. The kidney and brain have very similar vascular environments^22^, including low vascular resistance, high BP fluctuations, and many common risk factors^23^. Therefore, in an individual with an internal environment in which end-organ damage such as endothelial dysfunction or lipohyalinosis may occur in the small vessels of the kidney, it may also occur in the brain and is sufficient to cause cSVD^22^. In fact, also our research team has reported on an association between the estimated glomerular filtration rate and cSVD^24^, and many other researchers have also shown a close association between renal function and cSVD^22,23,25^. Second, BUN may influence the pathological mechanism via neurohormonal effects^21^. When the sympathetic nervous system or renin–angiotensin–aldosterone system is activated, renal excretion decreases and water, sodium, and BUN reabsorption increase^21,26^. Because high sympathetic activity is one of the risk factors for cSVD, high BUN may also identify a high-risk group for cSVD, regardless of renal function^27,28^. In fact, compared to creatinine and creatinine-to-albumin ratio, which reflect relatively pure renal function, BUN and BAR showed a closer association with cSVD (Supplementary Table 1). Last, high BUN levels can indicate severe conditions in which renal reabsorption must be promoted. However, our study population comprised relatively young and healthy individuals, and excluded patients with more severe conditions. Therefore, these factors may have a limited influence.

Furthermore, the resulting association between BAR and cSVD may be affected by low albumin levels. First, low albumin levels are associated with endothelial dysfunction. Albumin is a representative negative acute phase reactant whose synthesis decreases and catabolism increases under systemic inflammation^15,19^. In fact, as shown in Table 2, BAR showed a close correlation with other inflammatory markers (e.g., WBC counts and hs-CRP). Chronic inflammation damages the vascular endothelium and, by extension, the blood–brain-barrier (BBB)^29^. A damaged BBB causes periventricular infiltration of toxic metabolites and impairs their clearance through the glymphatic pathway, resulting in damage to the surrounding nervous tissue^1,30^. Therefore, hypoalbuminemia can be used as a biomarker for cSVD development and exacerbation due to endothelial dysfunction. Some studies have reported that albumin also plays a role in endothelial stabilization, supporting our hypothesis^14,31^. Second, albumin acts as an antioxidant that removes up to 70% of free radicals from serum^14,19^. Oxidative stress and free radicals are well-known risk factors for cSVD^32,33^; thus, a reduced antioxidant effect can also be a pathological mechanism of cSVD. Third, albumin can prevent microthrombus formation and the occlusion of perforating arterioles by inhibiting platelet activation or aggregation^19^. Thus, a decrease in albumin level may cause an increase in the cSVD subtypes of this ischemic mechanism (e.g., WMH and lacunes). Last, a decrease in albumin level can induce systemic hypoperfusion by reducing the osmotic pressure of the plasma, thereby reducing the circulating volume. However, our study population mostly included young and healthy individuals, and the albumin level was not low enough to induce significant hypoperfusion; therefore, this effect was not expected to be significant.

Most participants in our study had normal BUN and albumin levels. Perhaps because of this, each of the BUN and albumin levels showed statistical significance only in cSVD subtypes and univariate analysis, and did not show significance in multivariable analysis. However, BAR clearly showed an association with all cSVD subtypes. These findings suggest that although BUN and albumin levels are within known normal ranges, abnormal ratios between them may be associated with pathology that predisposes to cSVD. However, the threshold for BAR to predict high-risk groups for cSVD has not yet been determined. Further studies with extensive data are required to determine a clinically applicable cut-off value.

Our study has some limitations. First, this was a retrospective-cross sectional study. Although a close association between BAR and cSVD was observed, this does not guarantee a causal relationship. Second, we did not have data on the diets of the participants. A high-protein diet and dehydration are some of the factors that increase BUN; however, our registry does not contain nutritional information. As both BUN and albumin can be affected by recent diet, a single BAR value may not be representative in participants with large changes in recent diet or nutritional status. Third, the study population was relatively young and had few underlying diseases. Therefore, the influence of the well-known vascular risk factors may have been underestimated. Last, we performed an analysis using only one BAR value that was measured during the health check-up. cSVD is a chronic subclinical pathology that develops and worsens over several years. Therefore, it is difficult for a single BAR value to represent the underlying pathology. A more scientifically meaningful interpretation would have been possible if the analysis has been based on the average or change in the BAR values measured at various points in time.

In conclusion, we demonstrated that high BAR values were positively associated with all cSVD subtypes in health check-up participants. BAR has the potential to become a convenient, economical, and simple biomarker for cSVD as it can be measured using a simple blood test. However, these results and interpretations of our findings need to be validated through additional follow-up studies.

## Methods

### Study population

This study included consecutive health check-up participants who underwent brain MRI between January 2006 and December 2013 from the registry of the Seoul National University Hospital Health Promotion Center. Participants were excluded based on the following criteria: (1) age < 30 years; (2) history of stroke or severe neurological disease; (3) missing data for BUN, albumin, or major variables; and (4) malignancy, severe hepatic or renal disease. Finally, 3012 health check-up participants were included in the analyses.

The Institutional Review Board (IRB) of Seoul National University Hospital approved this study (No. 1502-026-647). The requirement for informed consent was waived by the IRB because of the retrospective study design and the use of de-identified information. All data covered in this study are presented in the manuscript and supplemental materials. All experiments were performed in accordance with the Declaration of Helsinki and the relevant guidelines.

### Major variables

We conducted extensive evaluations across the domains of demographic, clinical, and laboratory factors^34^. These included age, sex, BMI, hypertension, diabetes, hyperlipidemia, ischemic heart disease, current smoking, and systolic and diastolic BP^30,34^. Laboratory evaluations were performed after overnight fasting or fasting for at least 12 h. These included glucose profiles, lipid profiles, WBC counts, and hs-CRP, BUN, creatinine, GFR, and albumin levels^30,34^. GFR was calculated according to the Modification of Diet in Renal Disease (MRDR) equation. BAR was calculated using the following formula based on BUN and albumin levels: BAR = BUN (mg/dl)/albumin (g/dl)^13^.

All the study participants had undergone brain MRI and magnetic resonance angiography (MRA) using 1.5-T MR scanners on the day of the health check-up (Signa, GE Healthcare, Milwaukee, WI, USA or Magnetom, SONATA, Siemens, Munich, Germany)^30^. The detailed information of the MRI acquisitions is described as follows: basic slice thickness = 5 mm, T1-weighted images [repetition time (TR)/echo time (TE): 500/11 ms], T2-weighted images (TR/TE: 5000/127 ms), T2 fluid-attenuated inversion recovery images (TR/TE = 8800/127 ms), T2-gradient echo images (TR/TE = 57/20 ms), and 3-dimensional time-of-flight MRA images (TR/TE = 24/3.5 ms). The WMH volume was quantitatively measured using the Medical Imaging Processing, Analysis, and Visualization software (MIPAV, version, 11.0.0, National Institutes of Health, Bethesda, MD, USA) program, as in our previous studies^30^. For analysis, each participant's MRI data were obtained in the form of a DICOM file and entered into the program. The boundary of the WMH lesion was then delineated on the T2 fluid-attenuated inversion recovery images, and the volume was calculated using a semi-automated method by combining them for each slice^30^. Lacunes were defined as well-defined asymptomatic lesions of 3–15 mm in the territories of perforating arterioles with the same signal characteristics as cerebrospinal fluid on T1- or T2-weighted images^4^. CMBs were defined as focal round lesions < 10 mm in size with low-signal intensity on T2-gradient echo images^14^. Burdens of lacunes and CMBs were divided into absent, single, and multiple according to their numbers. All radiological parameters were rated by two well-trained neurologists (K.-W.N. and H.-Y.J.), and disagreements were resolved by discussion with a third investigator (H.-M.K.).

### Statistical analysis

Continuous variables with normal distributions are presented as arithmetic mean ± standard deviation, and those without a normal distribution are presented as median [interquartile range]. Continuous variables with skewed data, except for the WMH volume, were transformed to a log scale. The WMH volume had many zero values; therefore, it was transformed into a squared-root scale.

BAR is a parameter that can be somewhat unfamiliar. Therefore, by comparing the baseline characteristics of the participants according to the BAR value, we identified the characteristics of those with high BAR. For this analysis, the Kruskal–Wallis, Jonckheere–Terpstra, and Chi-square tests were used.

Univariate and multivariable analyses were used to identify possible predictors of cSVD. Simple linear regression analysis was used for WMH volume and univariate logistic regression analysis was used for lacunes and CMBs. Variables with *P* < 0.10 in the univariate analysis were included in the subsequent multivariable linear and logistic regression analyses along with BMI and GFR. If multicollinearity was highly suspected, variables considered more clinically relevant were included in the multivariable analysis model (e.g., systolic and diastolic BP). To assess the quantitative relationship between BAR and burden of cSVD, we compared BAR values according to burden for each cSVD pathology. For this purpose, the Jonckheere–Terpstra test was used. In addition, to determine the underlying pathophysiology between BAR and cSVD, we compared the strength of the association between BAR and cSVD with that between related parameters and cSVD. All statistical analyses were performed using SPSS version 23.0 (IBM Corp., Armonk, NY, USA). All variables with P < 0.05 were considered statistically significant.

### Supplementary Information

Supplementary Table 1.

## Data Availability

All data generated or analyzed during this study are included in this published article [and its supplementary information files].
