# Supplementary material for: Blood urea nitrogen to albumin ratio is associated with cerebral small vessel diseases
Source: Sci Rep. 2024 Feb 23;14:4455. doi: 10.1038/s41598-024-54919-8 (PMC10891149; doi:10.1038/s41598-024-54919-8)
Supplement: Supplementary file 1 — Supplementary Table 1. [file 41598_2024_54919_MOESM1_ESM.pdf]

Supplemental Materials

Supplemental Table I. Comparison of the association strength of BAR and related parameters for cSVD

	WMH volume	Lacunes	Cerebral microbleeds
	B (95% CI)	Adjusted OR [95% CI]	Adjusted OR [95% CI]
BUN to albumin ratio	0.068 (0.020 to 0.116)	1.20 [1.01-1.43]	1.26 [1.04-1.52]
Blood urea nitrogen	0.014 (0.003 to 0.025)	1.04 [1.00-1.08]	1.06 [1.01-1.10]
Albumin	-0.099 (-0.266 to 0.069)	0.55 [0.27-1.09]	1.25 [0.60-2.62]
Creatinine*	0.158 (-0.023 to 0.338)	1.82 [0.91-3.67]	1.69 [0.77-3.72]
Creatinine to albumin ratio*	0.191 (0.008 to 0.374)	2.13 [1.06-4.29]	1.62 [0.73-3.58]

WMH = white matter hyperintensity, BUN = blood urea nitrogen

*These variables were transformed into a log scale
